# Comparing the Efficacy of Electronically Delivered Cognitive Behavioral Therapy (e-CBT) to Weekly Online Mental Health Check-Ins for Generalized Anxiety Disorder—A Randomized Controlled Trial: Comparaison de l'efficacité de la thérapie cognitivo-comportementale délivrée par voie électronique (e-TCC) aux contrôles hebdomadaires en ligne de santé mentale pour le trouble d'anxiété généralisée - un essai randomisé contrôlé

**DOI:** 10.1177/07067437241261933

**Published:** 2024-07-21

**Authors:** Melinaz Barati Chermahini, Jazmin Eadie, Anika Agarwal, Callum Stephenson, Niloufar Malakouti, Niloofar Nikjoo, Jasleen Jagayat, Vineeth Jarabana, Amirhossein Shirazi, Anchan Kumar, Tessa Gizzarelli, Gilmar Gutierrez, Ferwa Khan, Charmy Patel, Megan Yang, Mohsen Omrani, Nazanin Alavi

**Affiliations:** 1 Department of Public Health Sciences, Faculty of Health Sciences, 4257 Queen's University, Kingston, Ontario, Canada; 2 Department of Psychiatry, Faculty of Health Sciences, 4257 Queen's University, Kingston, Ontario, Canada; 3 Department of Psychology, Faculty of Arts and Sciences, 4257 Queen's University, Kingston, Ontario, Canada; 4 Centre for Neuroscience Studies, Faculty of Health Sciences, 4257 Queen's University, Kingston, Ontario, Canada; 5 OPTT Inc., Toronto, Ontario, Canada

**Keywords:** mental health, generalized anxiety disorder, online cognitive behavioural therapy, eHealth, internet, electronic care, check-in, virtual, anxiety, psychotherapy, Santé mentale, trouble d’anxiété généralisée, thérapie cognitivo-comportementale en ligne, e-santé, internet, soins électroniques, contrôle, anxiété virtuelle, psychothérapie

## Abstract

**Background:**

Generalized anxiety disorder (GAD) is a prevalent anxiety disorder characterized by uncontrollable worry, trouble sleeping, muscle tension, and irritability. Cognitive behavioural therapy (CBT) is one of the first-line treatments that has demonstrated high efficacy in reducing symptoms of anxiety. Electronically delivered CBT (e-CBT) has been a promising adaptation of in-person treatment, showing comparable efficacy with increased accessibility and scalability. Finding further scalable interventions that can offer benefits to patients requiring less intensive interventions can allow for better resource allocation. Some studies have indicated that weekly check-ins can also lead to improvements in GAD symptoms. However, there is a lack of research exploring the potential benefits of online check-ins for patients with GAD.

**Objective:**

This study aims to investigate the effects of weekly online asynchronous check-ins on patients diagnosed with GAD and compare it with a group receiving e-CBT.

**Methods:**

Participants (*n* e-CBT = 45; *n* check-in = 51) with GAD were randomized into either an e-CBT or a mental health check-in program for 12 weeks. Participants in the e-CBT program completed pre-designed modules and homework assignments through a secure online delivery platform where they received personalized feedback from a trained care provider. Participants in the mental health check-in condition had weekly asynchronous messaging communication with a care provider where they were asked structured questions with a different weekly theme to encourage conversation.

**Results:**

Both treatments demonstrated statistically significant reductions in GAD—7-item questionnaire (GAD-7) scores over time, but when comparing the groups there was no significant difference between the treatments. The number of participants who dropped out and baseline scores on all questionnaires were comparable for both groups.

**Conclusions:**

The findings support the effectiveness of e-CBT and mental health check-ins for the treatment of GAD.

**Plain Language Summary Title:**

Comparing the Effectiveness of Electronically Delivered Therapy (e-CBT) to Weekly Online Mental Health Check-ins for Generalized Anxiety Disorder—A Randomized Controlled Trial

## Plain Language Summary

Generalized anxiety disorder (GAD) is a prevalent psychiatric condition that leads to symptoms like uncontrollable worry, trouble sleeping, muscle tension, and irritability. Cognitive behavioural therapy (CBT) is a common psychotherapy used for GAD since it has been shown to reduce symptoms. However, traditional CBT that is in person can have barriers such as being inaccessible and costly, and therefore electronically delivered CBT (e-CBT) is a viable alternative since previous studies have shown its efficacy in reducing symptoms and being similar compared to face-to-face CBT. Previous studies have also shown reductions in GAD symptomology through the use of checking in on people and their mental health. Therefore, this study aimed to compare e-CBT to a check-in condition and had a total of 45 individuals in e-CBT and 51 participants in the check-in condition. Participants in the e-CBT condition completed 12 weeks of predesigned e-CBT modules, homework and received personalized feedback from a care provider. In contrast, individuals in the check-in condition completed 12 weeks of unstructured asynchronous messaging with a care provider. Results from the study showed that both the e-CBT and check-in condition demonstrated statistically significant improvements in GAD-7 across time, but when comparing the groups there was no significant difference. The results show the efficacy of e-CBT and checking in on people's mental health to reduce GAD and future research should examine the 2 conditions combined.

## Résumé

**Contexte:**

Le trouble d’anxiété généralisée (TAG) est un trouble anxieux prévalent qui se caractérise par une inquiétude incontrôlable, des troubles du sommeil, des tensions musculaires et de l'irritabilité. La thérapie cognitivo-comportementale (TCC) est l’un des traitements de première intention qui a démontré une efficacité élevée pour réduire les symptômes d’anxiété. La TCC délivrée électroniquement (e-TCC) a été une adaptation prometteuse du traitement en personne, montrant une efficacité comparable avec une accessibilité et une évolutivité accrues. Trouver d'autres interventions évolutives pouvant offrir des avantages aux patients nécessitant des interventions moins intensives peut permettre une meilleure allocation des ressources. Certaines études ont indiqué que des contrôles hebdomadaires peuvent également entraîner une amélioration des symptômes du TAG. Cependant, il y a un manque de recherche explorant les avantages potentiels des enregistrements en ligne pour les patients souffrant du TAG.

**Objectif:**

Cette étude vise à étudier les effets des enregistrements asynchrones hebdomadaires en ligne sur les patients diagnostiqués avec un TAG et à les comparer à un groupe recevant l’e-TCC.

**Méthodes:**

Les participants (n e-TCC = 45 ; n enregistrement = 51) souffrant du TAG ont été randomisés dans un programme d'e-TCC ou un programme d'enregistrement de santé mentale pendant 12 semaines.. Les participants au programme de modules préconçus d’e-TCC et de devoirs via une plateforme de livraison en ligne sécurisée où ils ont reçu des commentaires personnalisés d'un prestataire de soins qualifié. Les participants à la condition d'enregistrement de santé mentale ont eu une communication hebdomadaire par messagerie asynchrone avec un prestataire de soins où on leur a posé des questions structurées avec un thème hebdomadaire différent pour encourager la conversation.

**Résultats:**

Les deux traitements ont démontré des réductions statistiquement significatives des scores TAG-7 au fil du temps, mais en comparant les groupes, il n'y avait aucune différence significative entre les traitements. Le nombre de participants ayant abandonné et les scores de base sur tous les questionnaires étaient comparables pour les deux groupes.

**Conclusions:**

Les résultats soutiennent l’efficacité de l’e-TCC et des contrôles de santé mentale pour le traitement du TAG

## Introduction

Over 450 million people live with generalized anxiety disorder (GAD), characterized by symptoms including uncontrollable worry most days, trouble sleeping, muscle tension, poor concentration, and irritability.^1–4^ Individuals with GAD can experience challenges in aspects of life including, social relationships, work, and school.^5^ Furthermore, GAD has a high comorbidity with depression, alcohol use disorder, and other anxiety disorders, making finding effective treatment options essential to reduce the prevalence of the other disorders.^6,7^

Cognitive behavioural therapy (CBT) is one of the first-line interventions for GAD, demonstrating high efficacy in symptom reduction compared to treatment as usual, waitlist controls, and other forms of psychotherapy at 6-month follow-ups.^8^ CBT targets thought patterns and behavior, allowing one to become more aware of their maladaptive thoughts which can aid in behavior change.^9^ CBT can effectively reduce symptoms of worry with comparable efficacy to pharmaceutical interventions.^10^ However, traditional face-to-face CBT poses accessibility barriers including long waitlists to see practitioners, high costs, and the stigma that can surround seeking in-person help.^11–14^

To address these barriers, electronically delivered CBT (e-CBT) is a promising solution and has been shown to improve symptoms of anxiety, depression, and sleep quality.^15–19^ e-CBT is also effective in treating GAD for a multitude of populations including older adults and postsecondary students.^20–22^ Moreso, e-CBT can provide comparable efficacy to its in-person counterpart.^23^ Given the widespread nature of electronic devices, e-CBT presents a unique opportunity to expand access to provide effective care. In a systematic review, smartphone app-based e-CBT significantly reduced anxiety symptoms but treatment effects were not as great compared to in-person CBT.^17,24^ Furthermore, e-CBT has been found to reduce social and recreational risk avoidance in people with GAD.^25^ However, one study showed that e-CBT was ineffective as a preventive method for reducing anxiety symptoms, but was still effective in improving secondary outcomes such as anxiety sensitivity.^26^ Additionally, e-CBT is more cost-effective than in-person and hybrid models.^27^ Therefore, finding a balance between cost-effectiveness and high-efficacy treatments is essential for proper resource allocation.

Checking in on people's mental health is a low-resource-intensive way to provide care and support, which can have positive effects on mental health.^28^ However, checking in on an individual's mental health with specific weekly topics through messaging has minimal research evidence supporting its efficacy. A study from New Zealand found that text messages directed at participants with depression and anxiety were a good alternative to face-to-face therapy, improving symptoms while also offering accessibility benefits and reducing fear of face-to-face interactions.^29^ Furthermore, text messaging services have become a popular way for therapists to communicate with their clients since they are accessible and convenient for different populations.^30^ However, text messaging check-in limitations include the inability to teach thoroughly constructed CBT. Therefore, using a more structured digital platform for mental health check-ins could address this limitation.^31,32^ More research on the effectiveness of online structured check-ins is needed.

## Objectives

This study investigated the efficacy of e-CBT compared to asynchronous online mental health check-ins for treating GAD. Based on evidence supporting the effectiveness of e-CBT, it was hypothesized that asynchronous online check-ins through weekly prompts would be effective at improving generalized anxiety disorder—7-item questionnaire (GAD-7) symptoms, but e-CBT would be superior.

## Methods

### Design

In this randomized trial, participants were randomly assigned through a randomization code in Excel, to either a 12-week e-CBT program or a mental health check-in program that used asynchronous online check-ins. After completing the check-in program, participants were given the option to voluntarily engage in the e-CBT program. Similarly, those who finished the e-CBT program could choose to participate in the check-in program. However, if participants decided to engage in the alternate afterwards, their data from the second program was not used for analysis. This study was registered through the ClinicalTrials.gov system (NCT04754438).

### Sample Size and Recruitment

Participants (total: *n* = 96; e-CBT: *n* = 45; check-in: *n* = 51) were recruited in Kingston, Ontario, Canada from outpatient psychiatry clinics at Kingston Health Sciences Centre, referrals from primary care physicians, specialists, clinicians, and self-referrals. Data from prior research that used this e-CBT program demonstrated that a sample of 50 participants in each group was sufficient for an effect size of 0.5, an α of .05, and a power of 0.8.^33^ Although 120 participants were recruited, only 96 started either the e-CBT or check-in condition.

### Eligibility

Inclusion criteria included being 18 years and older, a GAD diagnosis confirmed through a video call with a psychiatrist using the Diagnostic and Statistical Manual of Mental Disorders, 5th Edition,^34^ competence to consent to participate, consistent and reliable internet, and the ability to communicate and read English. Exclusion criteria included active psychosis, acute mania, suicidal/homicidal ideation, and severe alcohol/substance use disorder. Participants were also excluded if they received another psychotherapy program or received CBT in the past year to avoid confounding factors. Informed consent included disclosing the potential harms of the study and informing clients of their right to stop at any time. Once informed consent was obtained from all eligible participants, they were included in the study and randomly assigned to treatment groups by the lab manager.

### Interventions

*e-CBT*: Participants in the e-CBT arm were offered 12 weekly previously validated modules that focused on developing constructive and balanced coping strategies, behaviour modification, cognitive restructuring, relaxation strategies, and symptom management, which have all been shown to help reduce GAD.^16,19,35^ As seen in Table 1, the e-CBT modules also helped connect one's thoughts to emotions, physiological reactions, situations, and behaviours to become better engaged in day-to-day activities and learn to deal with negative situations or thoughts more productively. The weekly set of modules consisted of ∼30 slides that were delivered through the Online Psychotherapy Tool (OPTT), a secure, cloud-based, digital mental health platform (www.optt.health). Once participants finished their weekly module, they completed homework and submitted it to their care provider for personalized feedback. For each session of e-CBT, it takes ∼30 to 45 min to read the information and complete the homework, depending on the amount of effort that the client puts into their work. Each session was considered completed when the client finished and submitted their homework questions.

**Table 1.** Twelve-Week e-CBT Program Content and the Check-in Program Question Prompts.

**e-CBT session**	**e-CBT session description**
1. What is generalized anxiety disorder?	Provides expectations for the course and introduces anxiety and CBT.
2. The 5-part model	Introduces the concept of the 5-part model and how a situation, thoughts, feelings, physical reactions, and behaviours are connected and how they interact.
3. Strategies for stressful situations	Provides an overview of helpful strategies that can be used in stressful situations including pleasurable activities and helpful breathing techniques.
4. Situation, thoughts, feelings, physical reactions, and behaviours	Provide a further detailed exploration of the 5-part model and how changes in one area can affect the other 4 parts.
5. The thought record	Highlights the first 3 columns of the thought record; a tool used to help understand the connection between feelings, behaviours, and thoughts. The first 3 columns include the situation, followed by the feelings and automatic thoughts associated with it.
6. Automatic thoughts	This delves into the role of automatic thoughts and how they influence feelings. The focus of this session is to understand how to identify automatic thoughts and specifically identify the most dominant idea, or “hot thought” when presented with a stressful situation. Common thinking errors are also discussed in this session.
7. Activity scheduling	Provides a break from learning about the thought record and instead explains how to use an activity record; a tool designed to record and plan weekly activities. This session focuses on how tracking activities can inform mood changes and reinforce the scheduling of pleasurable activities.
8. Evidence	Focuses on the 4th and 5th columns of the thought record, which is designed to help gather the information that supports or does not support the identified hot thought.
9. Alternative and balanced thinking	Focuses on the final 2 columns of the thought record which reflects on the evidence columns to help find an alternative or balanced view of the situation. The last column invites the viewer to re-rate their feelings based on the completion of the thought record.
10. Experiments	Explain the importance of conducting experiments to start believing alternative or balanced thoughts from the thought record and initiating changes in ineffective thinking patterns.
11. Action plans	Centred on identifying a problem that needs to be solved and providing a framework for creating a plan for solving the problem.
12. Review	The final session is a review of the course and summarizes the main CBT concepts and tools that have been taught throughout the program.
**Check-in week**	**Check-in question prompts**
1. Mood	Tell me about yourself. How have you been feeling lately? How has the pandemic affected your mood? What differences have you noticed in your mood compared to the past?
2. Sleep	How have you been? Tell me about your week. I want to ask about your sleep. How many hours do you get? Do you feel rested? Do you think you need to make any changes to your sleep hygiene?
3. Activity	How was your week? How has your anxiety level been? What do you usually do to stay active during the day? Are there any indoor activities you can do at your place? Have you been using any online exercise programs?
4. Hobbies	What did you do in the past week? Do you have any hobbies? How long have you been doing them? Have you found any new activities you have enjoyed recently?
5. Friendship	How was your week? How has the pandemic affected your relationship with your friends? Have you stayed in touch with your close friends during the pandemic? How often do you interact with friends?
6. New events	How have you been? Have there been any new events in the past weeks? Are you planning to do anything new this week? Are you planning to do anything new this week? Are you expecting to hear any news from your family/friends?
7. Job, study	How have you been? Tell me about your week. How are you managing your day-to-day responsibilities (e.g., job, school, personal, etc.)?
8. Diet, food	How was your week? Let's talk about your eating habits. How does your anxiety affect your appetite? Are you satisfied with your diet? How often do you cook? Do you prefer homemade food or take-out?
9. Books, movies, television	How was your week? Has your anxiety affected your concentration? Do you like reading books? What is the last book you read? Are there any books you would recommend to your friends? What shows do you enjoy watching? What are some shows you would recommend to your friends?
10. Phone, apps, games	Tell me about your week. How many hours a day do you usually spend on your phone? What is your favourite app? Do you play any games? What kinds of games do you like to play (e.g., board games, cards, video games, etc.)?
11. Habits	How have you been? How was your week? What do you think are some of your healthy habits? What are some habits you would like to change?
12. Accomplishments	How was your week? How did you find this program? Were the weekly check-ins helpful? Overall, was this program beneficial for you? Why or why not? Are you interested in an online CBT program? Why or why not?

*Note*. CBT = cognitive behavioural therapy; e-CBT = electronically delivered CBT; DASS-42 = depression anxiety stress scale—42 item.

*Check-In*: Participants in the check-in arm had weekly check-ins with their care provider for 12 weeks through the asynchronous messaging system on OPTT. Care providers wrote to the participants weekly using structured pre-designed templates with questions (Table 1) and participants would communicate back to them. These questions were structured to encourage responses and therefore required the use of probing and critical thinking to help the clients better connect their feelings and experiences to the specific questions. Asking probing questions is a common therapy technique and has been shown to help individuals provide more details about the situation, feelings, and needs.^36^ Although care providers checked in on the participants and provided support, no psychotherapy strategies were taught. Therefore, it would take clients a few minutes to complete the check-in condition each week, but would depend on how much detail they wanted to use in order to answer the question. Each week, the check-in condition was considered completed when a client responded to the prompts that they were sent.

### Care Providers

The care providers in this study were research assistants who were medical doctors, with certifications in psychotherapy and also had training (mandatory CBT workshops) from the principal investigator (PI), who is an expert in the delivery of psychotherapy and online psychotherapy. They received training in e-CBT and learned how to provide feedback before interacting with participants. Their training included examining feedback scripts with practice participants and completing feedback that was reviewed by the PI. The PI or the other senior psychotherapist on the team (expert on online CBT) reviewed all weekly feedback before being submitted to participants. Care providers were given templates to write their feedback and personalized it based on participants’ homework submissions. The goal was to increase scalability similar to previous work showing that feedback tools can decrease the duration of writing feedback per participant, without sacrificing the quality of care.^35^

### Outcomes and Analysis

Participants completed reliable and clinically validated questionnaires at baseline, midpoint (week 6), and posttreatment (week 12). Participants completed the GAD-7, quality of life enjoyment and satisfaction questionnaire—short form (Q-LES-Q-SF), depression anxiety stress scale—42 item (DASS-42), and a demographic questionnaire.^37–40^ The GAD-7 was the primary outcome measure in order to examine changes in anxiety symptomatology. Secondary outcomes included Q-LES-Q-SF which assesses changes in quality of life and the DASS-42 which examines changes in depression, anxiety, and stress which are reported as such throughout.^37–40^

Before analysis, data were examined for missing, nonsensical, and outlying variables. Missing data were not imputed and was analysed on a per-protocol basis. The statistical significance level was 0.05, except in cases where a Bonferroni correction was used. Demographic information of program completers and dropouts was compared, and any possible differences were identified using independent samples *t*-tests. The primary analysis was intention-to-treat (ITT) conducted to determine the effect of treatment type on primary and secondary outcomes for all participants. A linear mixed-effects model was the main analysis used, with random effects as patient identification and fixed effects as treatment, time, and their interaction. This analysis involved all participants, including those who did not complete all sessions. Additionally, a 2 × 3 (treatment group × time) repeated measures analysis of variance (ANOVA) was performed on the primary and secondary outcomes. This analysis investigated changes in outcomes among individuals who completed all sessions of the treatments. The analyses were all done through IBM SPSS Statistics for Mac, Version 24 (IBM Corp., Armonk, NY, USA).

## Results

### Participants

**Figure 1.**
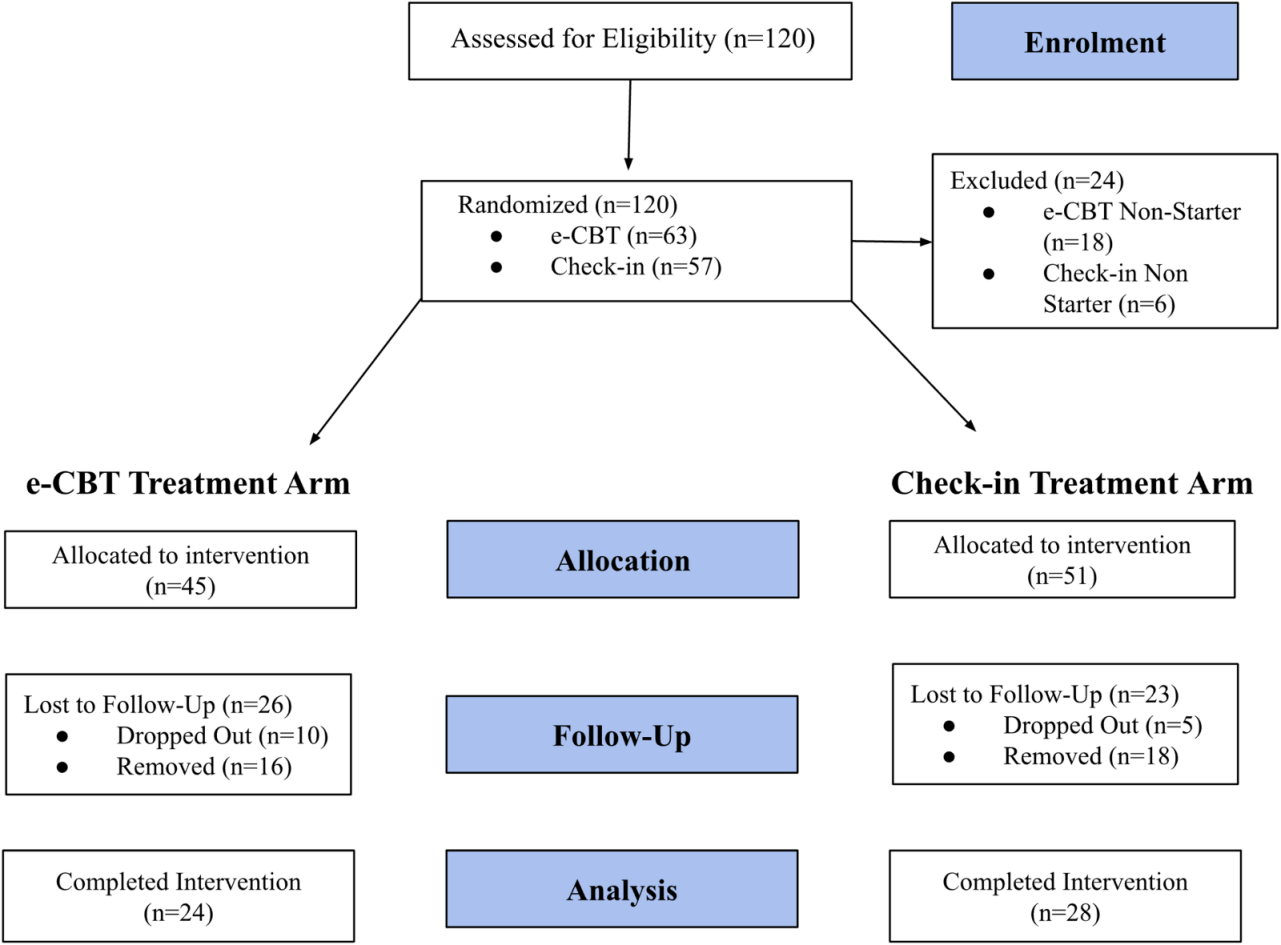
Participant recruitment and enrolment flowchart.

Recruitment occurred from May 2021 to August 2023 with a satisfactory sample size (Figure 1). Participants who began the interventions were included in the analysis and nonstarters were excluded, yielding 45 participants for e-CBT and a sample of 51 in the check-in condition. Across the e-CBT and check-in group, participants were an average of 34.5 (standard error (SE) = 1.36) years old, predominantly female (*n* = 65; 67.71%), and completed an average of 9.25 sessions (SE = 0.54). More specifically, participants in the e-CBT group completed an average of 8.68 (SE = 1.88) sessions compared to the check-in group who completed 9.85 (SE = 0.53) on average. In total 24 (53.33%) participants completed e-CBT and 28 (54.90%) completed check-in.

### Treatment Comparison: ITT Analysis

There was no difference in GAD-7 scores at baseline between the groups (e-CBT = 13.18, SE = 0.66; check-in = 13.55; SE = 0.59; *p* = 0.68). Q-LES-Q-SF scores were also similar (e-CBT = 40.38, SE = 1.48; check-in = 38.53; SE = 1.11; *p* = 0.32), and there was no significant difference in baseline depression (e-CBT = 18.80, SE = 1.82; check-in = 18.40, SE = 1.44; *p* = 0.89), anxiety (e-CBT = 16.50, SE = 1.35; check-in = 14.82, SE = 1.16; *p* = 0.35), or stress (e-CBT = 23.22, SE = 1.27; check-in = 21.80, SE = 1.11; *p* = 0.40) scores.

To compare the effect of treatment types on primary and secondary outcomes, an unstructured mixed-effect model was conducted (arm and time point as fixed factors), including participants who did not complete all treatment sessions (ITT analysis).

**Figure 2.**
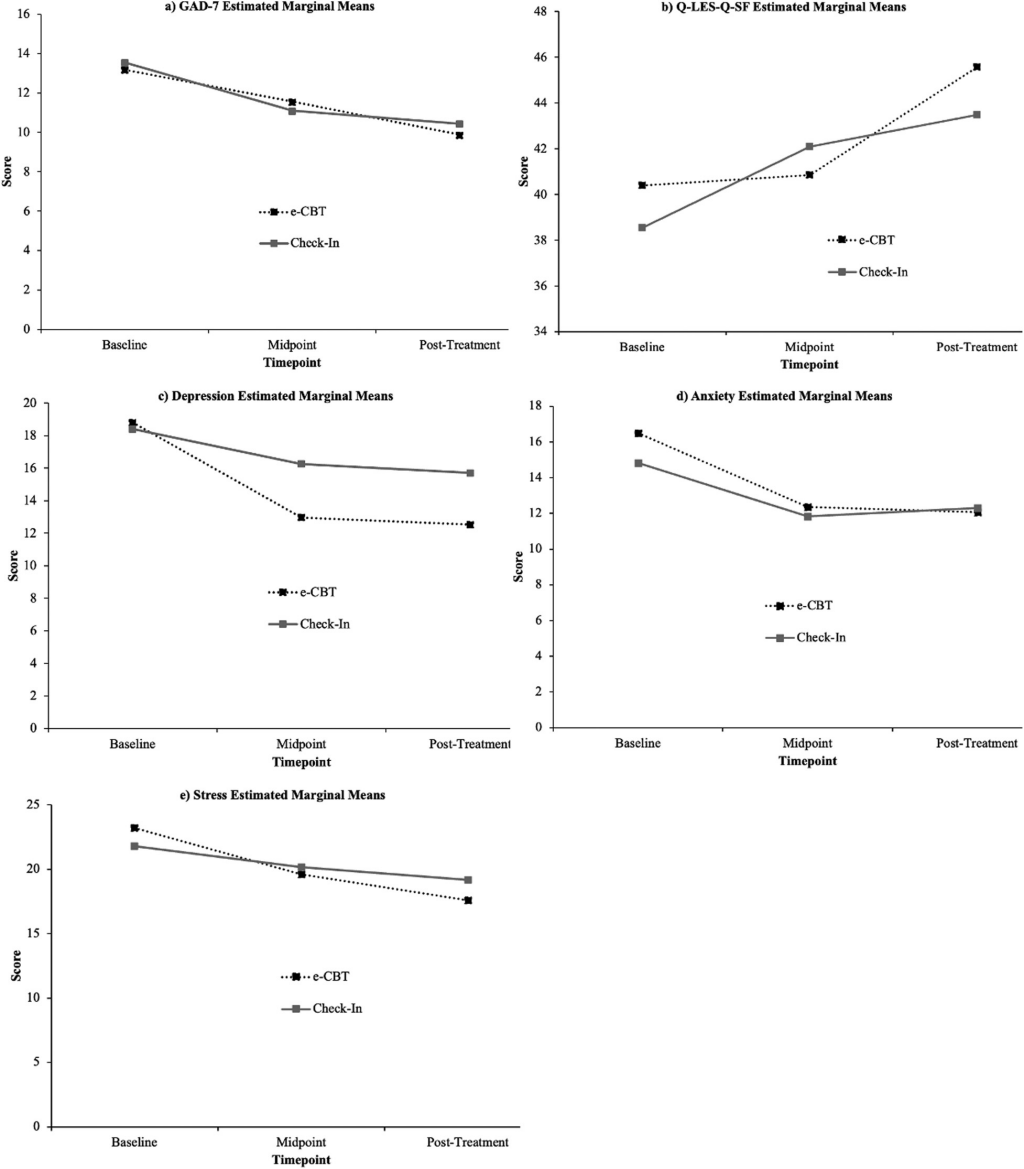
(a) GAD-7 scores at 3 points of treatment, initial (week 0), mid (week 6), and final (week 12), for e-CBT and check-in. (b) Q-LES-Q-SF scores at 3 points of treatment, initial (week 0), mid (week 6), and final (week 12), for e-CBT and check-in. (c) Depression (DASS-42) scores at 3 points of treatment, initial (week 0), mid (week 6), and final (week 12) in e-CBT and check-in. (d) Anxiety (DASS-42) scores at 3 points of treatment, initial (week 0), mid (week 6), and final (week 12), for e-CBT and check-in. (e) Stress (DASS-42) scores at 3 points of treatment, initial (week 0), mid (week 6), and final (week 12), in e-CBT and the check-in condition.

*Note*. GAD-7 = generalized anxiety disorder—7-item questionnaire; e-CBT = electronically delivered cognitive behavioural therapy; DASS-42 =  depression anxiety stress scale—42 item; Q-LES-Q-SF = quality of life enjoyment and satisfaction questionnaire—short form.

The GAD-7 scores for both e-CBT and check-in treatment groups significantly changed over time (*F* = 13.92, *p* < 0.001). The GAD-7 mean score (observed mean) for the e-CBT group decreased from 13.18 (baseline) to 11.56 (midpoint) to 9.875 (posttreatment), while the check-in scores decreased from 13.55 (baseline) to 11.1 (midpoint) to 10.44 (posttreatment). There was no significant difference between the treatment groups for GAD-7 scores (*F* = 0.024, *p* = 0.878). Moreover, the effect size representing the interaction of treatment and time was not significant (*F* = 1.368, *p* = 0.262; Table 2 and Figure 2a).

**Table 2.** Observed Means, SEs, and Linear Mixed Model Analysis of Primary and Secondary Outcomes as a Function 2 × 3 Design (Treatment Group × Time).

		Initial			Mid			Final			Initial to final	Outcome analysis—linear unstructured mixed models					
Questionnaire	Arm	*N*	*M*	SE	*N*	*M*	SE	*N*	*M*	SE	Effect size	Time		Treatment		Interaction	
											Cohen's *d*	*F*	*p*	*F*	*p*	*F*	*p*
GAD-7	e-CBT	44	13.1818	4.37898	32	11.5625	4.02362	24	9.8750	4.47517	0.32	13.922	<0.001	0.024	0.878	1.368	0.262
	Check-in	51	13.5490	4.23468	40	11.1	4.55620	27	10.4444	4.50071	−0.52						
											0.65						
											−0.59						
											−0.89						
Q-LES-Q-SF	e-CBT	39	40.3846	9.21559	26	40.8462	7.30311	23	45.5652	8.79476	0.29	5.358	0.008	0.801	0.373	2.461	0.095
	Check-in	49	38.5306	7.75699	37	42.0811	9.82734	19	43.4737	11.57281	−0.57						
											0.36						
											−0.28						
											−0.51						
Depression	e-CBT	44	18.7955	12.08801	30	12.9667	9.28285	23	12.5217	9.01952	0.44	5.484	0.006	0.487	0.487	1.102	0.339
	Check-in	50	18.4000	10.17400	40	16.2500	10.38181	27	15.7037	10.89042	−0.43						
											0.35						
											0.01						
											−0.56						
Anxiety	e-CBT	44	16.5000	8.94557	30	12.3667	7.26581	25	12.0800	8.93924	0.38	8.442	<0.001	0.464	0.497	0.397	0.674
	Check-in	51	14.8235	8.26730	39	11.8205	7.54241	27	12.2963	8.14680	−0.24						
											0.20						
											−0.01						
											−0.55						
Stress	e-CBT	45	23.2222	8.52507	30	19.6000	7.08860	25	17.6000	7.12390	0.33	6.403	0.003	0.011	0.918	1.575	0.215
	Check-in	50	21.8000	7.85325	38	20.1842	9.46360	27	19.1852	8.95732	−0.63						
											0.49						
											−0.29						
											−0.47						

*Note*. SE = standard error; GAD-7 = generalized anxiety disorder—7-item questionnaire; Q-LES-Q-SF = quality of life enjoyment and satisfaction questionnaire—short form; e-CBT = electronically delivered cognitive behavioural therapy.

The Q-LES-Q-SF mean score (observed mean) significantly improved from 40.38 to 40.85 to 45.56 (baseline, midpoint, and posttreatment) in the e-CBT group and from 38.53 to 42.08 to 43.47 in the check-in group (*F* = 5.358, *p* = 0.008). There was no significant difference between the treatment groups for the Q-LES-Q-SF score (*F* = 0.801, *p* = 0.373). The effect size for the interaction between treatment and time effects was also not significant (*F* = 2.461, *p* = 0.095; Table 2 and Figure 2b).

The scores for the depression section of the DASS-42 showed significant changes over time for both the e-CBT and check-in treatment groups (*F* = 5.484, *p* = 0.006). The mean score (observed score) from the depression section of the DASS-42, for the e-CBT group decreased from 18.80 (baseline) to 13.00 (midpoint) to 12.52 (posttreatment), while the check-in scores decreased from 18.40 (baseline) to 16.25 (midpoint) to 15.70 (posttreatment). There was no significant difference between the treatment groups for depression (DASS-42) scores (*F* = 0.487, *p* = 0.487). Moreover, the effect size representing the interaction of treatment and time effects was not significant (*F* = 1.102, *p* = 0.339; Table 2 and Figure 2c).

The anxiety (DASS-42) scores for both e-CBT and check-in treatment groups significantly decreased over time (*F* = 8.442, *p* < 0.001). The anxiety (DASS-42) mean score (observed mean) for the e-CBT group decreased from 16.50 (baseline) to 12.40 (midpoint) to 12.10 (posttreatment), while the check-in scores changed from 14.82 (baseline) to 11.82 (midpoint) to 12.30 (posttreatment). There was no significant difference between the treatment groups for anxiety (DASS-42) scores (*F* = 0.464, *p* = 0.497). Moreover, the effect size representing the interaction of treatment and time effects was not significant (*F* = 0.397, *p* = 0.674; Table 2 and Figure 2d).

The stress (DASS-42) scores for both e-CBT and check-in treatment groups significantly decreased over time (*F* = 6.403, *p* = 0.003). The stress (DASS-42) mean score (observed mean) for the e-CBT group decreased from 23.22 (baseline) to 19.60 (midpoint) to 17.60 (posttreatment), while the check-in scores decreased from 21.80 (baseline) to 20.18 (midpoint) to 19.18. There was no significant difference between the treatment groups for stress (DASS-42) scores (*F* = 0.011, *p* = 0.918). Moreover, the effect size representing the interaction of treatment and time effects was not significant (*F* = 1.575, *p* = 0.215; Table 2 and Figure 2e).

### Treatment Comparison-Completer Sample

A repeated measures ANOVA was performed on the primary and secondary outcomes to analyse changes in scores among treatment completers. The analysis showed no significant difference between e-CBT and check-in scores for GAD-7 (*F* = 0.070, *p* = 0.793), depression (*F* = 1.289, *p* = 0.262), Q-LES-Q-SF (*F* = 0.328, *p* = 0.571), anxiety (*F* = 0.038, *p* = 0.847) and stress (*F* = 0.012, *p* = 0.912). Additionally, a significant change in scores over time was observed for all outcomes GAD-7 (*F* = 16.41, *p* < 0.001), anxiety (*F* = 4.120, *p* = 0.048), stress (*F* = 5.806, *p* = 0.020), and Q-LES-Q-SF (*F* = 9.734, *p* = 0.004), except depression (*F* = 4.120, *p* = 0.212). The interaction term for the effect of time and treatment type was not significant for any of the outcomes including GAD-7 (*F* = 0.259, *p* = 0.613), depression (*F* = 0.408, *p* = 0.526), anxiety (*F* = 0.437, *p* = 0.512), stress (*F* = 2.577, *p* = 0.115), and Q-LES-Q-SF (*F* = 0.790, *p* = 0.380; Table 3).

**Table 3.** Observed Means, SEs, and Repeated Measures ANOVA of Primary and Secondary Outcomes as a Function of 2 × 3 Design (Treatment Group × Time).

			Initial		Mid		Final		Outcome Analysis—ANOVA								
Questionnaire	Arm	*N*							Time			Treatment			Interaction		
			*M*	SE	*M*	SE	*M*	SE	*df*	*F, p*	*ηp^2^*	*df*	*F, p*	*ηp^2^*	*df*	*F, p*	*ηp^2^*
GAD-7	e-CBT	22	12.68	0.93	11.73	0.95	9.36	0.91	1	16.407, <0.001	0.263	1	0.070, 0.793	0.002	1	0.259, 0.613	0.006
	Check-in	26	13.19	0.85	10.81	0.87	10.62	0.83									
Q-LES-Q-SF	e-CBT	18	38.72	2.32	40.72	2.22	44.61	2.55	1	9.734, 0.004	0.223	1	0.328, 0.571	0.010	1	0.790, 0.380	0.023
	Check-in	18	40.44	2.32	44.89	2.22	43.72	2.55									
Depression	e-CBT	20	14.55	2.42	12.70	2.16	11.85	2.23	1	1.602, 0.212	0.034	1	1.289, 0.262	0.028	1	0.408, 0.526	0.009
	Check-in	27	16.59	2.08	15.93	1.86	15.70	1.92									
Anxiety	e-CBT	23	14.74	1.76	12.13	1.71	11.83	1.74	1	4.120, 0.048	0.079	1	.038, 0.847	0.001	1	0.437, 0.512	0.009
	Check-in	27	13.78	1.63	11.41	1.58	12.30	1.61									
Stress	e-CBT	23	22.04	1.70	19.35	1.68	17.43	1.71	1	5.806, 0.020	0.110	1	.012, 0.912	0.000	1	2.577, 0.115	0.052
	Check-in	26	20.38	1.60	19.65	1.58	19.46	1.61									

*Note*. ANOVA = analysis of variance; *M *= mean; SE = standard error; *df* = degrees of freedom; GAD-7 = generalized anxiety disorder—7-item questionnaire; Q-LES-Q-SF = quality of life enjoyment and satisfaction questionnaire—short form; e-CBT = electronically delivered cognitive behavioural therapy.

There was no significant difference in patient compliance across groups. A chi-square test indicated that the number of patients who completed all 12 sessions was comparable across the 2 groups (e-CBT = 53.33%; check-in = 54.90%; *p* = 0.459). An independent sample *t*-test demonstrated that among dropouts, there was no significant difference regarding the number of sessions completed across both treatment groups (*p* = 0.658). On average, drop-outs completed 6.66 (SE = 0.675) sessions in the e-CBT arm and 7.09 (SE = 0.702) sessions in the check-in arm.

## Discussion

This study compared a 12-week e-CBT program to an online mental health check-in program for GAD. All baseline scores were comparable among the e-CBT and check-in conditions. Both the e-CBT and Check-in condition experienced improved symptoms from baseline to the end of treatment and no harm occurred. Results from the linear mixed model demonstrated a statistically significant improvement in primary and secondary outcomes over time. Furthermore, the repeated measures ANOVA showed that both the e-CBT and check-in condition resulted in statistically significant improvements in all outcomes over time except depression, which was not statistically significant.

In line with previous research, the results of this study suggest that e-CBT is an effective treatment for GAD.^12–17^ Additionally, this study's findings suggest that weekly asynchronous check-ins can also help to reduce symptoms of GAD. This improvement could be due to the therapeutic relationship established through online communication with a healthcare provider. Additionally, these improvements highlight the value of the supportive aspect of therapy in the treatment of GAD. These findings could inform mental health care systems to better allocate resources and improve accessibility of care. For example, patients on the waitlist, or those who need less intense follow-up after their symptoms are more stabilized, could benefit from e-CBT and check-ins.

Research on the effectiveness of check-ins in helping to reduce GAD is limited. Therefore, this study is original since it compares e-CBT with mental health check-ins in treating GAD and provides evidence of the effectiveness of check-ins for patients who require less direct support.

One of the limitations of this study is that the dropout rates were relatively high, potentially introducing bias and impacting our findings. For instance, it is possible that those who completed the study could have had different characteristics compared to those who dropped out and are not an accurate representation of the general patient population. Thus, the improvement in symptomatology may be influenced by other factors not investigated in the study. The dropout rate is comparable to studies that explored the effectiveness of e-CBT.^35^ Studies investigating the dropout rate for CBT to treat anxiety disorders and other mental health conditions reported a lower rate, ranging from 16% to 26%.^41,42^ It is crucial to further investigate the dropout rate of e-CBT and factors that can contribute to patients not completing treatment. Furthermore, previous studies investigating clinically meaningful change in GAD-7 scores have found a change ranging from 4 to 7 points to be clinically significant.^43,44^ Among the participants, the mean GAD-7 score decreased from the initial session to the final session for the e-CBT group (3.3) and check-in group (3.11; Table 2). Although the observed improvements were statistically significant (Table 2), they may not be clinically meaningful, which future research could investigate. In addition, this study did not have a long-term follow-up evaluation which should also be explored in future research.

In conclusion, this study compared e-CBT and an online mental health check-in program, and they both demonstrated efficacy in improving all of the primary and secondary symptoms across time when looking at the intention to treat analysis. This means that when e-CBT is not available, check-in messaging could be an alternative. Further investigation should be done to understand the effectiveness of these treatments for varying GAD severity, and their impact when used in conjunction, and longer follow-ups should be applied to examine long-term impacts.

## Supplemental Material

sj-doc-1-cpa-10.1177_07067437241261933 - Supplemental material for Comparing the Efficacy of Electronically Delivered Cognitive Behavioral Therapy (e-CBT) to Weekly Online Mental Health Check-Ins for Generalized Anxiety Disorder—A Randomized Controlled Trial: Comparaison de l'efficacité de la thérapie cognitivo-comportementale délivrée par voie électronique (e-TCC) aux contrôles hebdomadaires en ligne de santé mentale pour le trouble d'anxiété généralisée - un essai randomisé contrôlé Supplemental material, sj-doc-1-cpa-10.1177_07067437241261933 for Comparing the Efficacy of Electronically Delivered Cognitive Behavioral Therapy (e-CBT) to Weekly Online Mental Health Check-Ins for Generalized Anxiety Disorder—A Randomized Controlled Trial: Comparaison de l'efficacité de la thérapie cognitivo-comportementale délivrée par voie électronique (e-TCC) aux contrôles hebdomadaires en ligne de santé mentale pour le trouble d'anxiété généralisée - un essai randomisé contrôlé by Melinaz Barati Chermahini, Jazmin Eadie, Anika Agarwal, Callum Stephenson, Niloufar Malakouti, Niloofar Nikjoo, Jasleen Jagayat, Vineeth Jarabana, Amirhossein Shirazi, Anchan Kumar, Tessa Gizzarelli, Gilmar Gutierrez, Ferwa Khan, Charmy Patel, Megan Yang, Mohsen Omrani and Nazanin Alavi in The Canadian Journal of Psychiatry

## References

[1] McHugh RK Whitton SW Peckham AD , et al. Patient preference for psychological vs. pharmacological treatment of psychiatric disorders: a meta-analytic review. J Clin Psychiatry. 2013;74(6):595-602. doi:10.4088/JCP.12r07757 23842011 PMC4156137

[2] Wittchen HU Jacobi F Rehm J , et al. The size and burden of mental disorders and other disorders of the brain in Europe 2010. Eur Neuropsychopharmacol. 2011;21(9):655-679. doi:10.1016/j.euroneuro.2011.07.018 21896369

[3] Koerner N Dugas MJ Savard P , et al. The economic burden of anxiety disorders in Canada. Can Psychol. 2004;45(3):191-201.

[4] Rowa Antony NM . Generalized anxiety disorder. In: Miklowitz DJ, Craighead E, Craighea L, editors. Psychopathology: history, diagnosis, and empirical foundations. Hoboken, NJ: John Wiley & Sons Inc; 2008. p. 78-114.

[5] Powell TJ Enright SJ . Anxiety and stress management. London, UK and New York, NY: Routledge; 2015.

[6] Wang X Lin J Liu Q , et al. Major depressive disorder comorbid with general anxiety disorder: associations among neuroticism, adult stress, and the inflammatory index. J Psychiatr Res. 2022;148:307-314. doi:10.1016/j.jpsychires.2022.02.013 35193034

[7] Smith JP Book SW . Comorbidity of generalized anxiety disorder and alcohol use disorders among individuals seeking outpatient substance abuse treatment. Addict Behav. 2010;35(1):42-45. doi:10.1016/j.addbeh.2009.07.002 19733441 PMC2763929

[8] Tolin DF . Is cognitive–behavioral therapy more effective than other therapies? A meta-analytic review. Clin Psychol Rev. 2010;30(6):710-720. doi:10.1016/j.cpr.2010.05.003. 20547435

[9] Padesky CA Greenberger D . The clinician’s guide to CBT using mind over mood. 2nd ed. New York, NY: Guilford Press; 2016.

[10] Borza L . Cognitive-behavioral therapy for generalized anxiety. Dialogues Clin Neurosci. 2017;19(2):203-208. 28867944 10.31887/DCNS.2017.19.2/lborza PMC5573564

[11] Musiat P Tarrier N . Collateral outcomes in e-mental health: a systematic review of the evidence for added benefits of computerized cognitive behavior therapy interventions for mental health. Psychol Med. 2014;44(15):3137-3150. doi:10.1017/S0033291714000245 25065947

[12] Czyz EK Horwitz AG Eisenberg D , et al. Self-reported barriers to professional help seeking among college students at elevated risk for suicide. J Am Coll Health. 2013;61(7):398-406. doi:10.1080/07448481.2013.820731 24010494 PMC3788673

[13] Wuthrich VM Frei J . Barriers to treatment for older adults seeking psychological therapy. Int Psychogeriatr. 2015;27(7):1227-1236. doi:10.1017/S1041610215000241 25739459

[14] Dew A Bulkeley K Veitch C , et al. Addressing the barriers to accessing therapy services in rural and remote areas. Disabil Rehabil. 2013;35(18):1564-1570. doi:10.3109/09638288.2012.720346 23009191

[15] Carl JR Miller CB Henry AL , et al. Efficacy of digital cognitive behavioral therapy for moderate-to-severe symptoms of generalized anxiety disorder: a randomized controlled trial. Depress Anxiety. 2020;37(12):1168-1178. doi:10.1002/da.23079 32725848

[16] Alavi N Stefanoff M Hirji A Khalid-Khan S . Cognitive behavioural therapy through PowerPoint: efficacy in an adolescent clinical population with depression and anxiety. Int J Pediatr. 2018;2018:1396216. doi:10.1155/2018/1396216 30532790 PMC6250002

[17] Soh HL Ho RC Ho CS Tam WW . Efficacy of digital cognitive behavioural therapy for insomnia: a meta-analysis of randomised controlled trials. Sleep Med. 2020;75:315-325. doi:10.1016/j.sleep.2020.08.020 32950013

[18] Gellatly J Chisnall L Seccombe X , et al. @Home etherapy service for people with common mental health problems: an evaluation. Behav Cogn Psychother. 2018;46(1):115-120. doi:10.1017/S1352465817000297 28506333

[19] Alavi N Hirji A . The efficacy of PowerPoint-based CBT delivered through email: breaking the barriers to treatment for generalized anxiety disorder. J Psychiatr Pract. 2020;26(2):89-100. doi:10.1097/PRA.0000000000000455 32134882

[20] Jones SL Hadjistavropoulos HD Soucy JN . A randomized controlled trial of guided internet-delivered cognitive behaviour therapy for older adults with generalized anxiety. J Anxiety Disord. 2016;37:1-9. doi:10.1016/j.janxdis.2015.10.006 26561733

[21] Newman MG Kanuri N Rackoff GN , et al. A randomized controlled feasibility trial of internet-delivered guided self-help for generalized anxiety disorder (GAD) among university students in India. Psychotherapy. 2021;58(4):591-601. doi:10.1037/pst0000383 34881930 PMC8744990

[22] Paxling B Almlöv J Dahlin M , et al. Guided internet-delivered cognitive behavior therapy for generalized anxiety disorder: a randomized controlled trial. Cogn Behav Ther. 2011;40(3):159-173. doi:10.1080/16506073.2011.576699. 21770848

[23] Carlbring P Maurin L Törngren C , et al. Individually-tailored, internet-based treatment for anxiety disorders: a randomized controlled trial. Behav Res Ther. 2011;49(1):18-24. doi:10.1016/j.brat.2010.10.002 21047620

[24] Linardon J Cuijpers P Carlbring P , et al. The efficacy of app-supported smartphone interventions for mental health problems: a meta-analysis of randomized controlled trials. World Psychiatry. 2019;18(3):325-336. doi:10.1002/wps.20673 31496095 PMC6732686

[25] Lorian CN Titov N Grisham JR . Changes in risk-taking over the course of an internet-delivered cognitive behavioral therapy treatment for generalized anxiety disorder. J Anxiety Disord. 2012;26(1):140-149. doi:10.1016/j.janxdis.2011.10.003 22079215

[26] Christensen H Batterham P Mackinnon A , et al. Prevention of generalized anxiety disorder using a web intervention, iChill: randomized controlled trial. J Med Internet Res. 2014;16(9):e199. doi:10.2196/jmir.3507 PMC4211086 25270886

[27] You JHS Luk SWC Chow DYW , et al. Cost-effectiveness of internet-supported cognitive behavioral therapy for university students with anxiety symptoms: a Markov-model analysis. PLOS ONE. 2022;17(5):e0268061. doi:10.1371/journal.pone.0268061 PMC9070891 35511888

[28] Harandi TF Taghinasab MM Nayeri TD . The correlation of social support with mental health: a meta-analysis. Electron Physician. 2017;9(9):5212-5222. doi:10.19082/5212 29038699 PMC5633215

[29] Anstiss D Davies A . “Reach out, rise up”: the efficacy of text messaging in an intervention package for anxiety and depression severity in young people. Child Youth Serv Rev. 2015;58:99-103. doi:10.1016/j.childyouth.2015.09.011

[30] Nolan C Quinn S MacCobb S . Use of text messaging in a mental health service for university students. Occup Ther Ment Health. 2011;27(2):103-125. doi:10.1080/0164212X.2011.565702.

[31] Wu MS Chen SY Wickham RE , et al. Outcomes of a blended care coaching program for clients presenting with moderate levels of anxiety and depression: pragmatic retrospective study. JMIR Ment Health. 2021;8(10):e32100. doi:10.2196/32100 PMC8569535 34673534

[32] Mohr DC Schueller SM Tomasino KN , et al. Comparison of the effects of coaching and receipt of app recommendations on depression, anxiety, and engagement in the IntelliCare platform: factorial randomized controlled trial. J Med Internet Res. 2019;21(8):e13609. doi:10.2196/13609 PMC6737883 31464192

[33] Alavi N Moghimi E Stephenson C , et al. Comparison of online and in-person cognitive behavioral therapy in individuals diagnosed with major depressive disorder: a non-randomized controlled trial. Front Psychiatry. 2023;14:1113956. doi:10.3389/fpsyt.2023.1113956 37187863 PMC10175610

[34] American Psychiatric Association. Diagnostic and statistical manual of mental disorders, fifth edition (DSM-5). American Psychiatric Association; 2013.

[35] Alavi N Omrani M . Online cognitive behavioral therapy: an e-mental health approach to depression and anxiety. Cham, Switzerland: Springer International Publishing; 2019. doi:10.1007/978-3-319-99151-1

[36] Wärdig R Engström AS Carlsson A Wärdig F Hultsjö S . Saving lives by asking questions: nurses’ experiences of suicide risk assessment in telephone counselling in primary health care. Prim Health Care Res Dev. 2022;23:e65. doi:10.1017/s146342362200055x. PMC9641664 36285522

[37] Barnes LLB Harp D Jung WS . Reliability generalization of scores on the Spielberger State-Trait Anxiety Inventory. Educ Psychol Meas. 2002;62(4):603-618. doi:10.1177/0013164402062004005

[38] Endicott J Nee J Harrison W Blumenthal R . Quality of life enjoyment and satisfaction questionnaire: a new measure. Psychopharmacol Bull. 1993;29(2):321-326. 8290681

[39] Spitzer RL Kroenke K Williams JBW , et al. A brief measure for assessing generalized anxiety disorder: the GAD-7. Arch Intern Med. 2006;166(10): 1092–1097. doi:10.1001/archinte.166.10.1092 16717171

[40] Imam SS . Depression anxiety stress scales (DASS): revisited. J Behav Sci. 2008;3(1):104-116.

[41] Bentley KH Cohen ZD Kim T , et al. The nature, timing, and symptom trajectories of dropout from transdiagnostic and single-diagnosis cognitive-behavioral therapy for anxiety disorders. Behav Ther. 2021;52(6):1364-1376. doi:10.1016/j.beth.2021.03.007. 34656192 PMC8531532

[42] Fernandez E Salem D Swift JK , et al. Meta-analysis of dropout from cognitive behavioral therapy: magnitude, timing, and moderators. J Consult Clin Psychol. 2015;83(6):1108-1122. doi:10.1037/ccp0000044 26302248

[43] Bischoff T Anderson SR Heafner J , et al. Establishment of a reliable change index for the GAD-7. Psychol Commun Health. 2020;8(1):176-187. doi:10.5964/pch.v8i1.309

[44] Toussaint A Hüsing P Gumz A , et al. Sensitivity to change and minimal clinically important difference of the 7-item generalized anxiety disorder questionnaire (GAD-7). J Affect Disord. 2020;265:395-401. doi:10.1016/j.jad.2020.01.032 32090765

